# ASB6 as an Independent Prognostic Biomarker for Colorectal Cancer Progression Involves Lymphatic Invasion and Immune Infiltration

**DOI:** 10.7150/jca.93066

**Published:** 2024-03-17

**Authors:** Qingyong Hu, Yahui Chen, Qianru Zhou, Shanshan Deng, Bo Mu, Jiancai Tang

**Affiliations:** Institute of Basic Medicine and Forensic Medicine, North Sichuan Medical College, Nanchong, 637000, China.

**Keywords:** ASB6, CRC, Bioinformatics, prognosis, immune infiltration, IP-MS

## Abstract

**Background:** ASB6, an E3 ubiquitin ligase, mediates the proteasomal degradation of its substrate proteins via the ubiquitin-proteasome pathway. ASB6 has been reported to play significant roles in several biological processes, including tumor stemness and endoplasmic reticulum stress. However, the underlying role and mechanism of ASB6 in colorectal cancer, particularly its association with immune infiltration levels and its prognostic significance, remain to be fully elucidated.

**Methods:** We identified key prognostic genes in CRC patients through LASSO-penalized Cox regression, Univariate and Multivariate Cox regression analyses. Subsequently, we comprehensively analyzed the prognostic value of hub genes and constructed a prognostic nomogram. Finally, we identified ASB6 interacting proteins through immunoprecipitation-mass spectrometry (IP-MS) and constructed protein-protein interaction (PPI) networks and performed pathway enrichment analysis to explore the potential mechanisms of ASB6. Meanwhile, we evaluated the functions of ASB6 in CRC cells through in vitro cell experiments.

**Results:** We identified ASB6 as a hub gene in CRC. ASB6 was highly expressed in CRC, and patients with high ASB6 expression had worse Disease-Free Interval (DFI), Disease-Specific Survival (DSS), Overall Survival (OS), and Progression-Free Interval (PFI). Correlation analysis showed that ASB6 expression were positively correlated with lymph node invasion and distal metastasis. Overexpression of ASB6 enhanced the migration ability of CRC cells. Multivariate Cox regression analysis revealed that ASB6 was an independent prognostic factor for OS and DSS in CRC. The nomogram model constructed based on multivariate analysis results had good predictive effects, with C-indexes of 0.811 and 0.934 for OS and DSS, respectively. Furthermore, analysis of immune infiltration levels showed that ASB6 expression were positively correlated with M2-type macrophage infiltration levels in CRC, and patients with high levels of both ASB6 and M2-type macrophages had a worse prognosis. Furthermore, pathway enrichment analysis of ASB6 interacting proteins identified by IP-MS suggested that ASB6 may play a crucial role through the response to unfolded protein pathway and protein processing in the endoplasmic reticulum pathway.

**Conclusions:** ASB6 is significantly upregulated in CRC tissues and is a risk factor for prognosis in CRC patients. ASB6 enhances the migration ability of CRC cells. Therefore, ASB6 may be an independent prognostic biomarker and potential therapeutic target for CRC patients.

## Introduction

Colorectal cancer is the third most common cancer and the second leading cause of cancer-related deaths worldwide 1. Colorectal cancer accounts for approximately 10% of the total annual diagnosed cancer cases and cancer-related deaths globally 2, 3. There are about 935,000 deaths from colorectal cancer and nearly 2 million new cases diagnosed each year worldwide 1. According to the latest cancer statistics from the National Cancer Center of China, colorectal cancer ranks third in incidence and fourth in mortality among common cancers, following lung cancer, female breast cancer, and lung cancer, liver cancer, and gastric cancer, respectively 4. With increasing cancer incidence and mortality, cancer has become a leading cause of death and poses a serious threat to the health and lives of people worldwide, making it a major global public health issue. Colorectal cancer exhibits high tumor heterogeneity and treatment typically involves a combination of surgery and chemotherapy based on disease grade, stage, and other characteristics 5, 6. With improvements in therapies and regimens, the 5-year survival rate of colorectal cancer patients has significantly improved to 65% 7, 8. However, treatment options and efficacy are limited for metastatic or advanced stages patients, and corresponding prognostic biomarkers are lacking. Therefore, improving diagnosis, treatment, and prevention of colorectal cancer, as well as further exploring the underlying molecular mechanisms and biological characteristics of colorectal cancer growth and progression are imperative to enhance the health and lives of people worldwide.

Ubiquitination and deubiquitination modifications are highly conserved post-translational modifications (PTMs), with ubiquitination being the second most common PTM of proteins after phosphorylation 9, 10. It plays a crucial role in numerous biological processes and diseases including cancer, involving proteasomal degradation of proteins, cell cycle, inflammation, apoptosis, DNA repair, stress response, and drug resistance 11-13. This process requires a three-step enzymatic cascade mediated by E1 ubiquitin-activating enzymes (E1s), E2 ubiquitin-conjugating enzymes (E2s), and E3 ubiquitin ligases (E3s) to covalently attach ubiquitin molecules to target proteins 14. E3s confer high substrate specificity and determine the fate of the substrate proteins 15. Meanwhile, ubiquitination is a dynamic and reversible process. Deubiquitinating enzymes remove ubiquitin chains from target proteins to prevent their degradation, thereby balancing the effects of ubiquitination 16. In mammalian cells, researchers have identified over 600 E3s and around 100 deubiquitinases (DUBs), which exhibit substrate specificity and regulate distinct cellular functions 17-19. As such, E3s and DUBs have emerged as promising potential drug targets for cancer therapy. Numerous studies have demonstrated that many E3s and DUBs are implicated in the initiation and progression of colorectal cancer 20-27. However, systematic investigation into the correlation between E3s and DUBs with the prognosis of colorectal cancer holds significant clinical importance for the identification of novel prognostic markers.

In this study, we employed bioinformatics analysis to systematically screen for E3s and DUBs genes significantly associated with the prognosis of colorectal cancer patients. Notably, we identified ASB6 (Ankyrin repeat and SOCS box protein 6) as a key prognostic gene in colorectal cancer. ASB6 belongs to the SOCS box protein superfamily and forms an SCF-like ECS (Elongin-Cullin-SOCS-box protein) E3 ubiquitin ligase complex with Elongin-Cullin, which mediates the proteasomal degradation of its substrate proteins via the ubiquitin-proteasome pathway 28. ASB6 has been reported to play significant roles in several biological processes. Previous studies have indicated that the upregulation of ASB6 induced by areca nut extracts is associated with the occurrence of betel quid-induced oral cancer 29. Moreover, researchers have found that ASB6 enhances stemness properties and maintains metastatic potential of oral squamous cell carcinoma cells by alleviating endoplasmic reticulum stress 30. Additionally, ASB6 functions as an effector protein, counteracting the circFNDC3B-mediated inhibition of stemness, and acts as an adaptor protein, facilitating circINSIG1-induced cholesterol biosynthesis in colorectal cancer 31, 32. These results all indicate that ASB6 may play a role in tumorigenesis and progression. However, there is currently limited research on the mechanisms and roles of ASB6 in cancer. Furthermore, there is a lack of systematic studies on ASB6 in pan-cancer, particularly in colorectal cancer. Consequently, we analyzed the expression levels of ASB6 in pan-cancer and its copy number variations, immune infiltrations, clinical correlations, and prognostic values in colorectal cancer. We also constructed a risk score nomogram to predict the survival of colorectal cancer patients. In sum, these results collectively indicate that ASB6 could serve as a novel prognostic marker and potential therapeutic target in colorectal cancer.

## Materials and methods

### LASSO-penalized Cox regression, Univariate and Multivariate COX regression Analysis

Clinical and mRNA-seq data for 473 colorectal cancer patients and 41 normal controls were retrieved from the TCGA database. After excluding cases lacking complete survival data, the analysis incorporated the mRNA expression and clinical data of 441 patients with colorectal cancer. Then, we utilized these data to perform univariate Cox analysis to assess the correlation between 1223 genes in the human ubiquitination and de-ubiquitination complex genes (UBDUB, From Addgene #171531) and overall survival (OS) prognosis. Subsequently, we performed LASSO-penalized Cox regression on genes with p-values less than 0.05 from the univariate Cox analysis to select prognostically relevant hub genes. Moreover, we further conducted multivariate Cox analysis on these hub genes to explore their independent prognostic significance.

### Pan-cancer differential expression, Clinicopathologic features, prognosis, and epigenetic analysis of ASB6

The datasets consisting of 33 cancer types from UCSC Xena (https://xenabrowser.net/datapages/) was utilized to analyze the differential expression of ASB6. The expression levels of ASB6 in normal and tumor tissues were visualized with box plots, and the significance of differential expression was analyzed using the Wilcox-test. The expression levels of ASB6 in colorectal cancer patient tissues with different clinical features are presented in box plots, with significance determined by Pearson's chi-squared test. The R packages "survminer" and "survival" were utilized to calculate the optimal cutoff values and perform survival analysis for OS, PFI, DSS, and DFI in COAD and READ, respectively.

We conducted an analysis of the DNA methylation levels of ASB6 in pan-cancer using the Gene Set Cancer Analysis (GSCA) web tool 33 (http://bioinfo.life.hust.edu.cn/GSCA/#/). Additionally, we evaluated the survival differences between ASB6 copy number alterations (CNA) statuses and the wild type in COAD based on CNA module. The cBioPortal web tool (https://www.cbioportal.org) was utilized to analyze the expression levels of ASB6 in various copy number alteration (CNA) subgroups in colorectal cancer 34. Furthermore, we also explored associations between ASB6 mRNA expression and CNA values or methylation levels.

### Establishment and validation of nomograms

Based on the results of univariate and multivariate Cox regression analyses for overall survival (OS) and disease-specific survival (DSS), we constructed separate nomograms for ASB6 alone or in combination with clinical variables having p-values less than 0.05 in the multivariate Cox analysis. We evaluated the predictive accuracy of the nomograms by calculating the concordance index (C-index) and plotting calibration curves.

### Cell culture, reagents, plasmids, and lentivirus infection

Human embryonic kidney HEK-293T cells and human colon cancer DLD-1 cells were generously gifted by Dr. Fengtian Li and Wenting Liao (Sun Yat-sen University Cancer Center, China). HEK-293T cells and DLD-1 cells were cultured in DMEM and RPMI-1640 medium (GIBCO, Rockville, MD, USA) supplemented with 10% fetal bovine serum (FBS, C04001, Biological Industries, Beit Haemek, Israel), 100 units/mL penicillin, and 100 μg/mL streptomycin (Penicillin-Streptomycin Liquid, P1400, Solarbio, China), respectively. The cells were maintained at 37°C in a 5% CO2 humidified incubator. Polybrene (C0351) and Puromycin (ST551) were purchased from Beyotime (Shanghai, China). Anti-Flag Magnetic Beads (HY-K0207) were purchased from MCE (Shanghai, China).

The plasmids pLVX-ASB6-Flag (ASB6-Flag) and lentiCRISPRv2-sgASB6 (sgASB6) used for recombinant lentiviral production were constructed in pLVX-puro and lentiCRISPRv2 vectors (kindly provided by Dr. Hu Chen 35 and our lab), respectively. All plasmids used in this study were confirmed by DNA sequencing. Lentiviral particles and lentiviral infection were performed as previously described 36. The primer sequences used for amplification and sgRNA are as follows:

For ASB6-Flag, Forward primer: 5'-CTACCGGACTCAGATCTCGAGGCCACCATGCCGTTCCTGCACGGCTTCC-3'; Reverse primer: 5'-GTCGCTGCCGCTGCCGAATTCGATGTCATCTTCCACGGAGCCACTG 3'. For lentiCRISPRv2-sgNC (sgNC), Forward primer: 5'-CACCGTGCGAATACGCCCACGCGAT-3'; Reverse primer: 5'-AAACATCGCGTGGGCGTATTCGCAC-3'. For lentiCRISPRv2-sgASB6, Forward primer: 5'-CACCGGTTCCTGCACGGCTTCCGG-3'; Reverse primer: 5'-AAACCCGGAAGCCGTGCAGGAACC-3'.

### Wound healing assays

DLD-1 cells stably expressing empty vector (EV), ASB6-Flag, non-targeting control single guide RNA (sgNC), or ASB6 knockout sgRNA (sgASB6) were seeded at a density of 500,000 cells per well in 12-well plates. After overnight incubation to allow cell adherence, linear scratches were generated in the confluent monolayers using a 200 μl pipette tip. Cells were washed with phosphate-buffered saline (PBS) to remove cellular debris, after which RPMI-1640 medium supplemented with 2% fetal bovine serum (FBS) was added. The positions of the scratches were observed under a microscope, photographed, and marked as 0 hour (h). Cells were then incubated at 37°C with 5% CO2 for 24 h, washed again with PBS, and imaged at the 24 h timepoint at the same marked scratch positions. Finally, cell migration distances were quantified and compared across groups.

### Western blot, and mass spectrometry

Western blot analysis was performed as previously described 36. The antibodies used in this study were as follows: Flag (1:1000, catalog #14793, Cell Signaling Technology (CST)), ASB6 (1:1000, catalog #21449-1-AP, proteintech), GAPDH (1:5000, catalog #60004-1-Ig, proteintech). For mass spectrometry, the supernatant was collected from stable DLD-1 colorectal cancer cells expressing ASB6-Flag or empty vector (EV) control, and then the supernatant was immunoprecipitated using magnetic beads coupled with Flag antibody. The proteins enriched by the anti-Flag Magnetic Beads were digested with trypsin and desalted, followed by identification analysis using mass spectrometry (LUMINGBIO, Shanghai, China). Finally, the raw data were analyzed by ProteomeDiscoverer 2.5 software and matched to a human protein database. Proteins with unique peptides identified in the ASB6-Flag sample but not in the EV control were considered as ASB6-interacting proteins.

### Immunohistochemistry (IHC) Analysis

Immunohistochemistry analysis was performed as previously described 36. Human colon adenocarcinoma tissue microarrays (HCol-Ade180Sur-04) were purchased from Outdo Biotech (Shanghai, China). The antibody for ASB6 (Catalog No. PA5-52077, dilution 1:200) was purchased from Invitrogen (Carlsbad, USA). For quantitative analysis, two experienced pathologists determined the scores based on the percentage of positive staining cells and staining intensity. The scoring system for the degree of positive staining categorized the results into five levels: <5% (score 0), 5%-25% (score 1), 26%-50% (score 2), 51%-75% (score 3), and >75% (score 4). Staining intensity was classified into four grades: no staining (score 0), weak staining (score 1), moderate staining (score 2), and strong staining (score 3). The final score was calculated by multiplying the score for positive percentage and staining intensity. Based on these scores, cases were segregated into high expression (scores >7) and low expression (scores ≤7) groups.

### Correlation Analysis

The TIMER database 37 (https://cistrome.shinyapps.io/timer/) was used to analyze the correlation between the expression levels of ASB6 and immune infiltration in colon cancer and rectal cancer (COAD and READ).

### PPI network construction and pathway enrichment analysis

The Metascape online tool (https://metascape.org/gp/index.html#/main/step1) was used for protein-protein interaction (PPI) network and pathway enrichment analysis 38. Default parameters were used, including a minimum overlap of 3, P-value cutoff of 0.01, and minimum enrichment of 1.5. The Molecular Complex Detection (MCODE) algorithm was then applied to detect densely connected network components in the enriched pathways. Gene Ontology (GO) and Kyoto Encyclopedia of Genes and Genomes (KEGG) enrichment analyses were performed using the ClusterProfiler R package.

### Statistical analysis

R (version 4.0.5) and the GraphPad Prism 8 software (GraphPad Software Inc., USA) were applied for statistical analysis. The correlation between the expression levels of ASB6 and clinicopathological characteristics of patients was analyzed using the Pearson's Chi-square test. Survival analysis was performed by the Kaplan-Meier (KM) method. The Wilcox-test method was used for comparing the expression levels of ASB6 in paired cancer and adjacent samples in the TCGA-COAD database, as well as comparing the expression levels of ASB6 between normal and tumor tissues in pan-cancer. The C-index was used to evaluate the predictive ability of the nomogram model. In addition to the statistical methods described above, the comparison between two groups was conducted using a two-tailed unpaired Student's *t*-test for significance testing. P values less than 0.05 were considered statistically significant.

## Results

### ASB6 is a prognostically relevant hub gene in COAD

**Figure 1:**
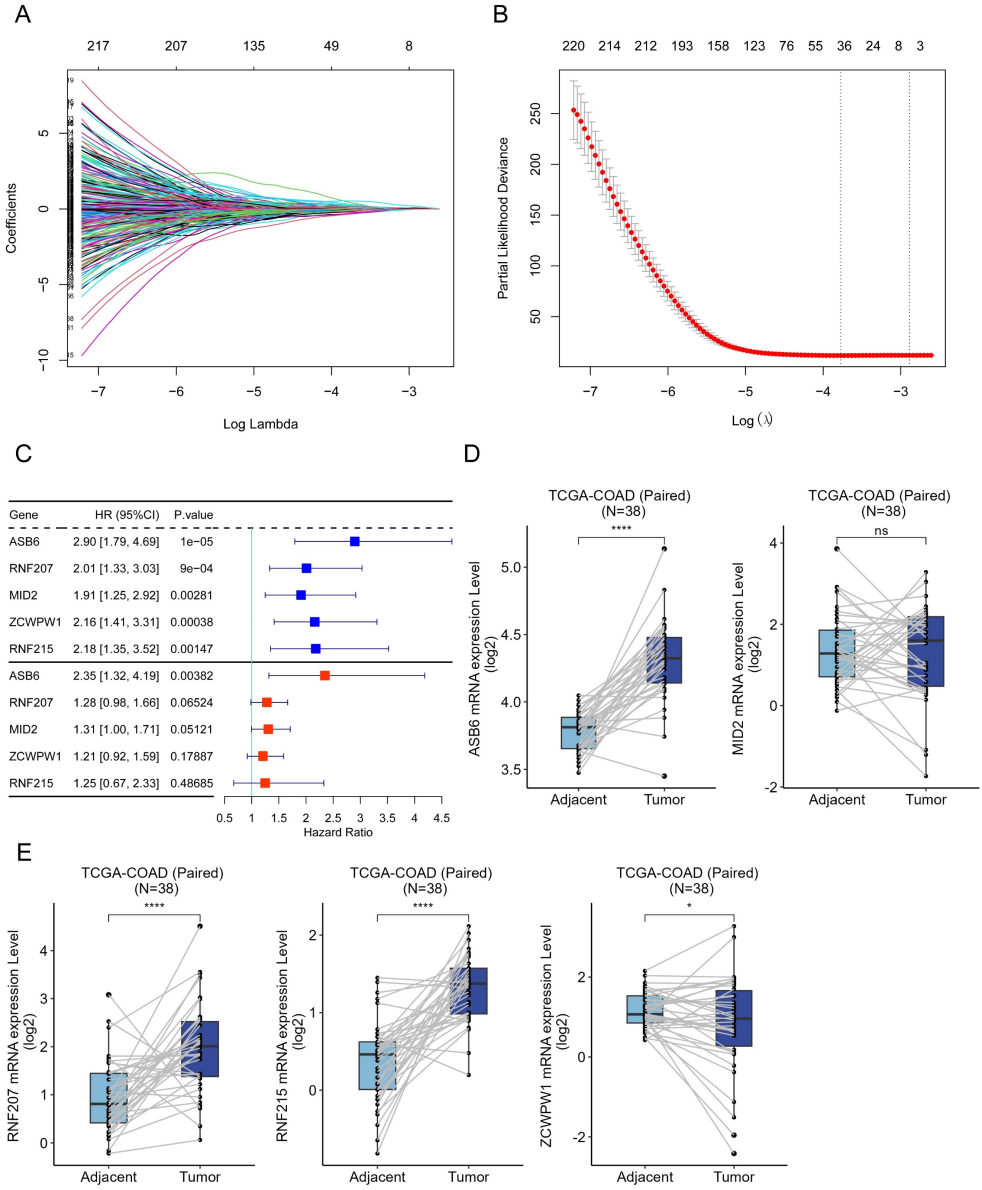
Construction of the UBDUB signature model. (A) Lasso coefficient profiles of the human ubiquitination and de-ubiquitination complex genes (UBDUB); (B) Analyzing the partial likelihood deviance of variables in the Lasso model using 10-fold cross-validation;(C) Forest plot showing the relationship between the expression of five genes obtained from Lasso model and overall survival (OS) (blue: Univariate analysis; red: Multivariate analysis);(D-E) The mRNA expression levels of five genes in tumor samples and paired adjacent normal tissues in the TCGA-COAD database; (*p < 0.05; **p < 0.01; ***p < 0.001; ****p < 0.0001; ns, Not Significant).

Ubiquitination and deubiquitination are important post-translational modifications of proteins, involved in various biological processes 11-13. Accordingly, we collected 1223 genes involved in ubiquitination and deubiquitination modifications from Addgene to identify prognostically relevant biomarkers. Initially, we performed univariate Cox analysis on these 1223 genes using colorectal cancer survival data, leading to the identification of 503 genes significantly associated with overall survival. Subsequently, we further analyzed these 503 genes using LASSO-penalized Cox regression with ten-fold cross-validation, which yielded a lambda.1se parameter of 0.0421 after cross-validation (Figure 1A, B). Ultimately, we identified ASB6, RNF207, MID2, ZCWPW1, and RNF215 as the five hub genes. Univariate and multivariate COX regression analyses showed that these five hub genes acted as risk factors in colorectal cancer, with ASB6 demonstrating the most significant association with prognosis (Univariate: HR=2.9, 95% CI: 1.79-4.69; Multivariate: HR=2.35, 95% CI: 1.32-4.19) (Figure 1C). Further analysis of the expression levels of these five hub genes in colorectal cancer and adjacent normal tissues revealed that ASB6, RNF207, and RNF215 were significantly upregulated in colorectal cancer tissues, while ZCWPW1 was significantly downregulated (Figure 1D, E). Taken together, these results suggest that ASB6 may serve as an independent prognostic factor in colorectal cancer.

### ASB6 is an independent prognostic factor in colorectal cancer

**Figure 2:**
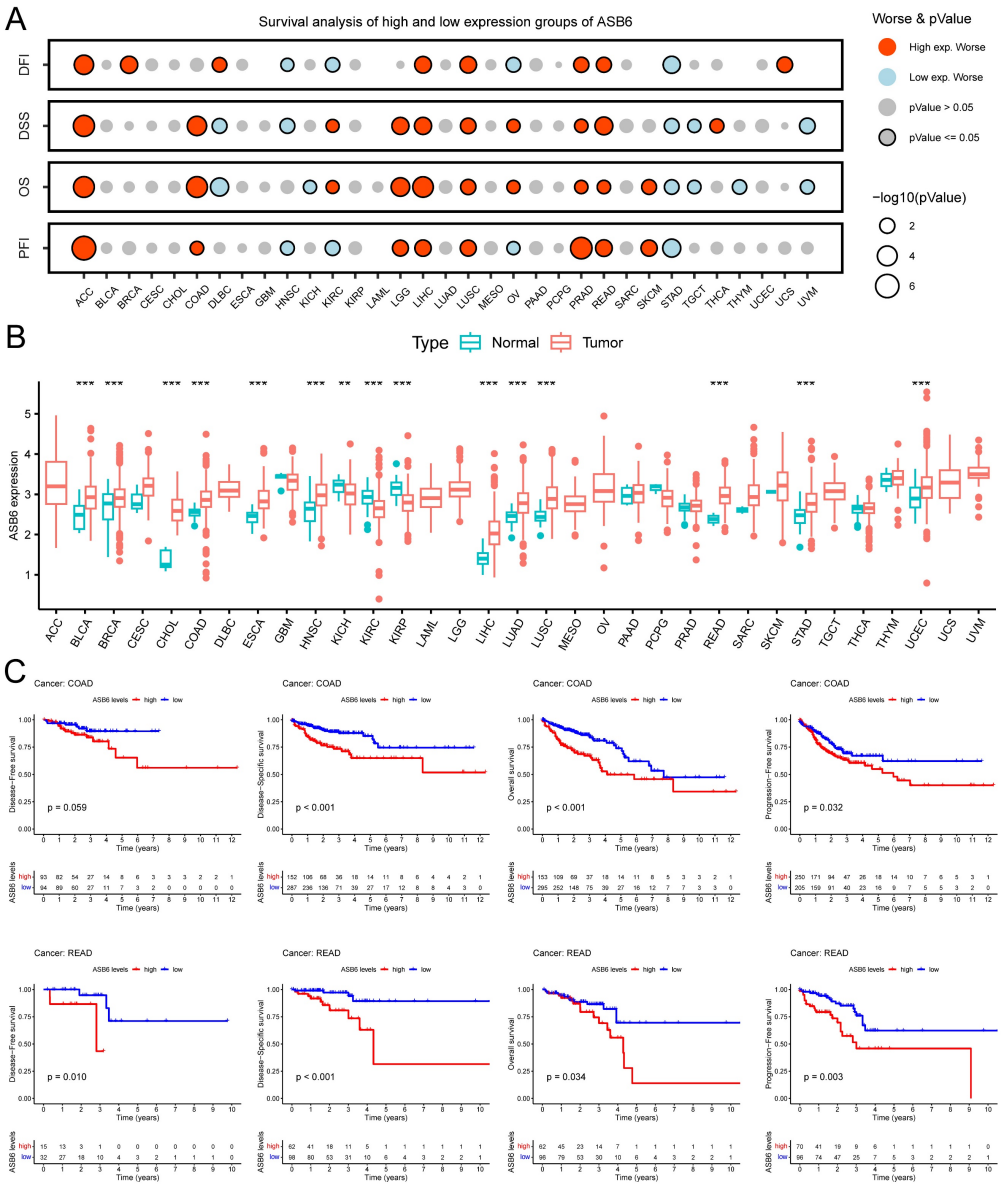
Pan-cancer analysis of ASB6. (A) The relationship between the expression of ASB6 and prognosis in pan-cancer. (B) Differences in the expression of ASB6 between the normal and tumor tissues in pan-cancer of TCGA datasets. (C) High expression of ASB6 was significantly correlated with shorter DFI, DSS, OS, and PFI of COAD and READ in TCGA datasets; (*p < 0.05; **p < 0.01; ***p < 0.001; ****p < 0.0001; ns, Not Significant).

Based on the analysis of survival data across 33 cancer types in TCGA and UCSC databases, we found that high expression of ASB6 was significantly associated with shorter disease-free interval (DFI), disease-specific survival (DSS), overall survival (OS), and progression-free interval (PFI) in adrenocortical carcinoma (ACC), liver hepatocellular carcinoma (LIHC), lung squamous cell carcinoma (LUSC), prostate adenocarcinoma (PRAD), and rectum adenocarcinoma (READ) (Figure 2A, C). Furthermore, high ASB6 expression correlated with shorter DSS, OS, and PFI in colon adenocarcinoma (COAD) and lower grade glioma (LGG) as well (Figure 2A, C). The differential expression analysis of ASB6 in pan-cancer also revealed significant upregulation in 12 cancer types (12/22, with 11 other types having too few or no normal samples), including the aforementioned cancers LIHC, LUSC, COAD, and READ (Figure 2B). Furthermore, the results of univariate and multivariate COX analysis of OS and DSS survival data for COAD in the TCGA database also showed that ASB6 can serve as an independent prognostic factor for COAD (Tables 1, 2).

### Upregulation of ASB6 is associated with advanced clinicopathological characteristics in COAD

To further elucidate the correlation between ASB6 expression levels and clinicopathological characteristics, we stratified colorectal cancer patient tissues into low and high ASB6 expression groups based on the optimal cutoff value determined by overall survival analysis. This enabled examination of the clinical implications of ASB6 expression levels. As shown in Table 3, ASB6 expression did not significantly correlate with age, gender, Kras mutation status, T stage (T3-T4 vs. T1-T2), N stage (N1+ vs. N0), history of colonpolyps, or pathological stage (III-IV vs. I-II). However, high ASB6 expression was significantly associated with lymphatic invasion and living status (all p < 0.01). Additionally, high ASB6 expression weakly correlated with M stage (M1 vs. M0) and cancer status (With tumor vs. Tumor-free).

**Figure 3:**
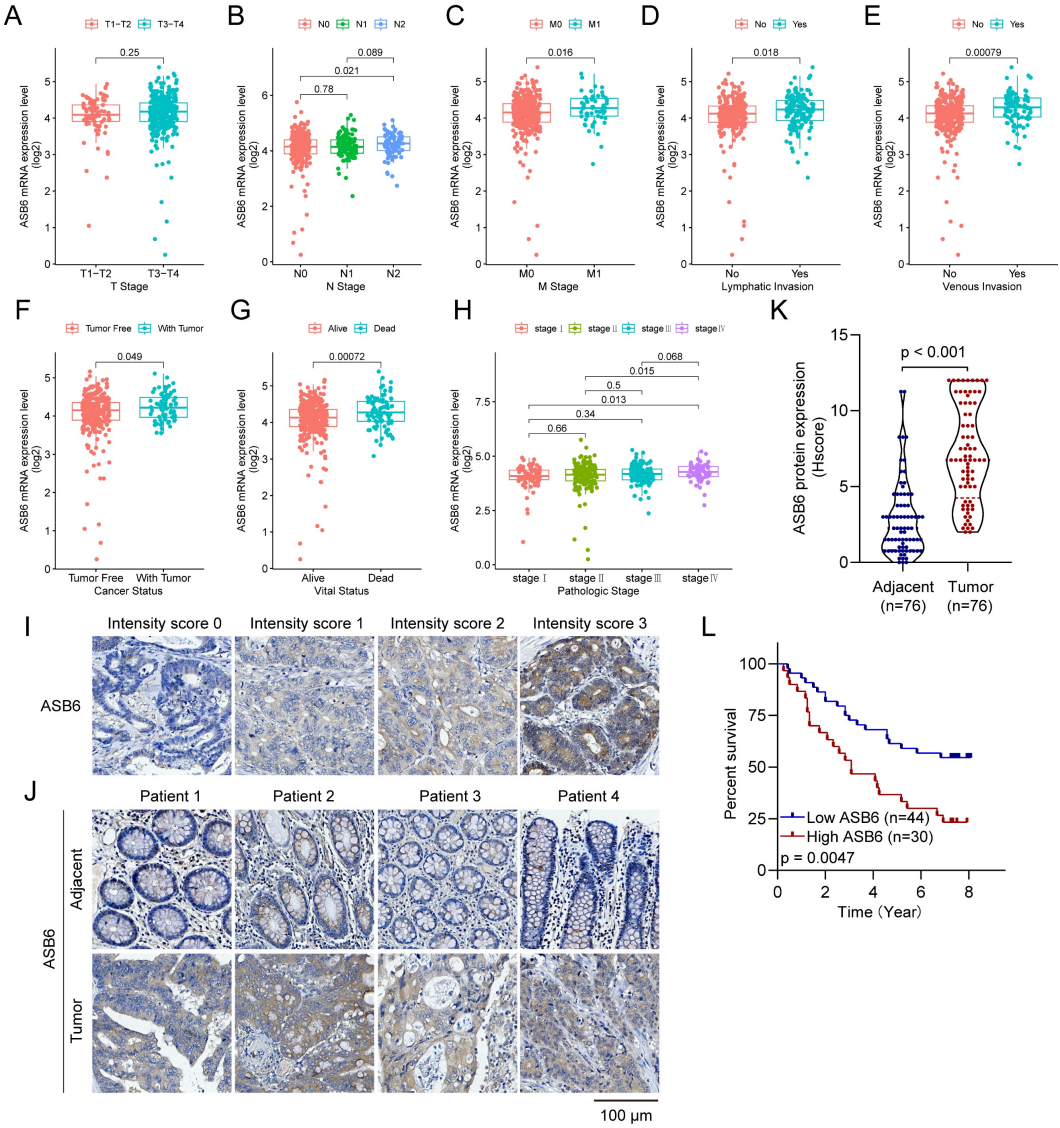
Analyzing ASB6 expression based on the clinicopathological characteristics of colorectal cancer patients in the TCGA dataset and examining the correlation between ASB6 protein expression levels and prognosis. (A) The expression levels of ASB6 were analyzed in T1-T2 and T3-T4 colorectal cancer patients. (B) The expression levels of ASB6 were analyzed in N0, N1, and N2 colorectal cancer patients. (C) The expression levels of ASB6 were analyzed in M0, and M1 colorectal cancer patients. (D) The expression levels of ASB6 were analyzed in colorectal cancer patients based on their lymphatic invasion status (Yes or No). (E) The expression levels of ASB6 were analyzed in colorectal cancer patients based on their venous invasion status (Yes or No). (F) The expression levels of ASB6 were analyzed in colorectal cancer patients based on their cancer invasion status (Tumor free or With tumor). (G) The expression levels of ASB6 were analyzed in colorectal cancer patients based on their vital status (Alive or Dead). (H) The expression levels of ASB6 were analyzed in colorectal cancer patients based on tumor pathological stages (stage I, II, III, and IV). (I) Staining intensity was classified into four grades: no staining (score 0), weak staining (score 1), moderate staining (score 2), and strong staining (score 3). (J) Representative micrographs of ASB6 protein expression levels in colorectal cancer and adjacent normal tissues. Scale bar =100 μm. (K) Quantitative analysis of the protein expression levels of ASB6 in colorectal cancer and adjacent normal tissues of the tissue microarray. Data were presented as mean ± SD. Comparisons were performed with two-tailed Student's *t*-test. (L) Patients with high protein levels of ASB6 had a poor prognosis. (*p < 0.05; **p < 0.01; ***p < 0.001; ****p < 0.0001; ns, Not Significant).

Additionally, we analyzed the expression levels of ASB6 among clinicopathological subgroups. We found ASB6 expression was significantly higher in N2 stage compared to N0 stage tissues (Figure 3B). Likewise, ASB6 expression was significantly elevated in M1 versus M0 stage tissues (Figure 3C). In colorectal cancer tissues with lymph node or vascular invasion, ASB6 expression was markedly higher relative to non-invaded tissues (Figure 3D, E). ASB6 levels were also significantly upregulated in patient tissues from those with persistent tumors or who were deceased compared to controls (Figure 3F, G). Moreover, pathological stage IV colorectal cancer tissues exhibited notably higher ASB6 expression than stage I and II patient tissues (Figure 3H). To further clarify the role of ASB6 in the prognosis of colorectal cancer patients, we analyzed the protein expression levels of ASB6 in colorectal cancer tissue microarrays through immunohistochemistry analysis. We found that, compared with adjacent normal tissues, the protein expression levels of ASB6 were significantly upregulated in colorectal cancer tissues, and patients with high protein levels of ASB6 had a poor prognosis (Figure 3I-L). Collectively, these results demonstrate ASB6 expression correlates with colorectal cancer progression, further confirming its potential as a biomarker for poor prognosis in colorectal cancer patients.

### Nomograms construction and validation based on the independent prognostic factors

**Figure 4:**
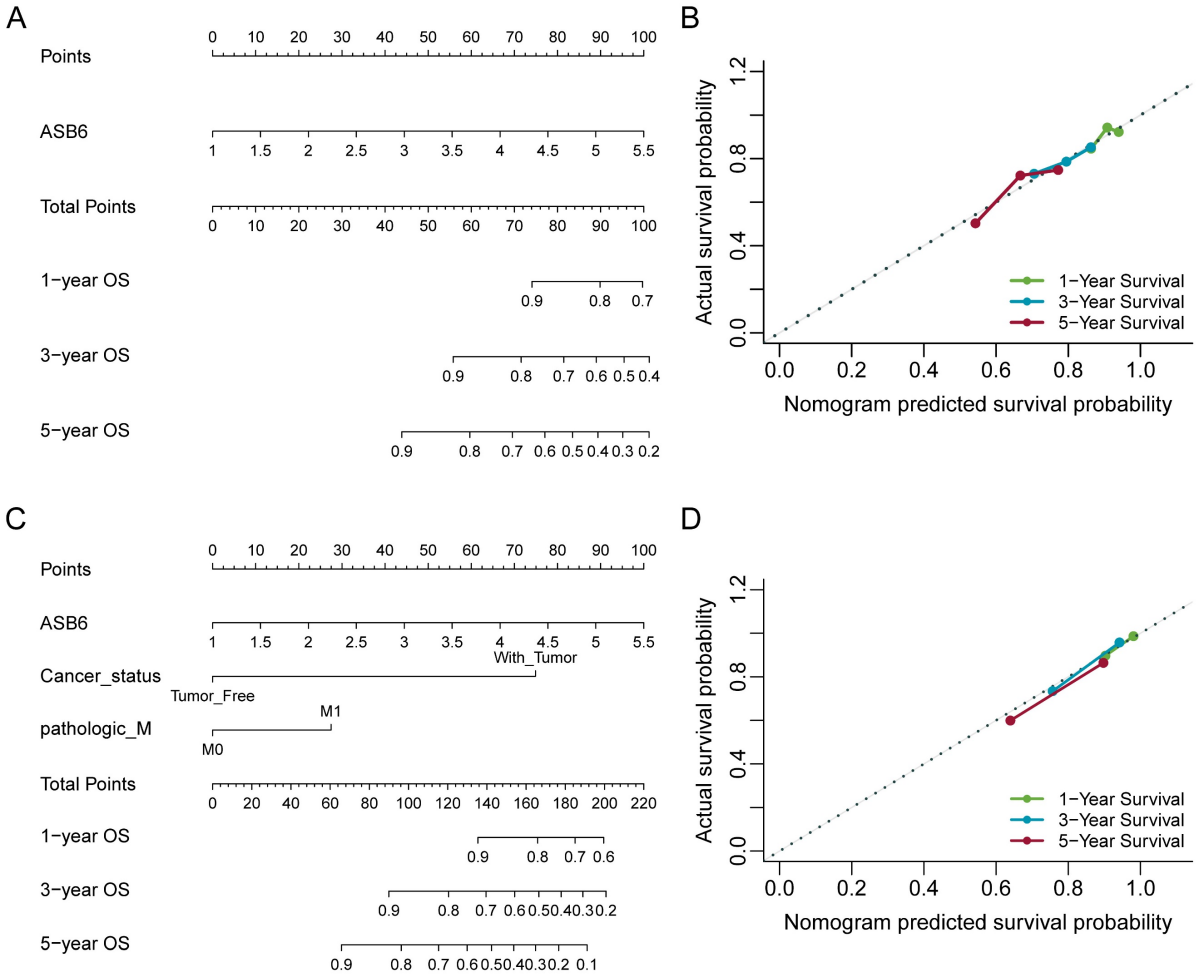
Nomogram for calculating risk score and predicting overall survival (OS) probability. (A, C) Postoperative prognostic nomogram for colorectal cancer patients was established based on ASB6 expression levels, cancer status, pathologic M; (B, D) Calibration curves of the nomogram were plotted to compare predicted and actual overall survival (OS) proportions at 1 year, 3 years, and 5 years. The x-axis represents the nomogram-predicted OS probability, and the y-axis represents the actual OS proportion.

**Figure 5:**
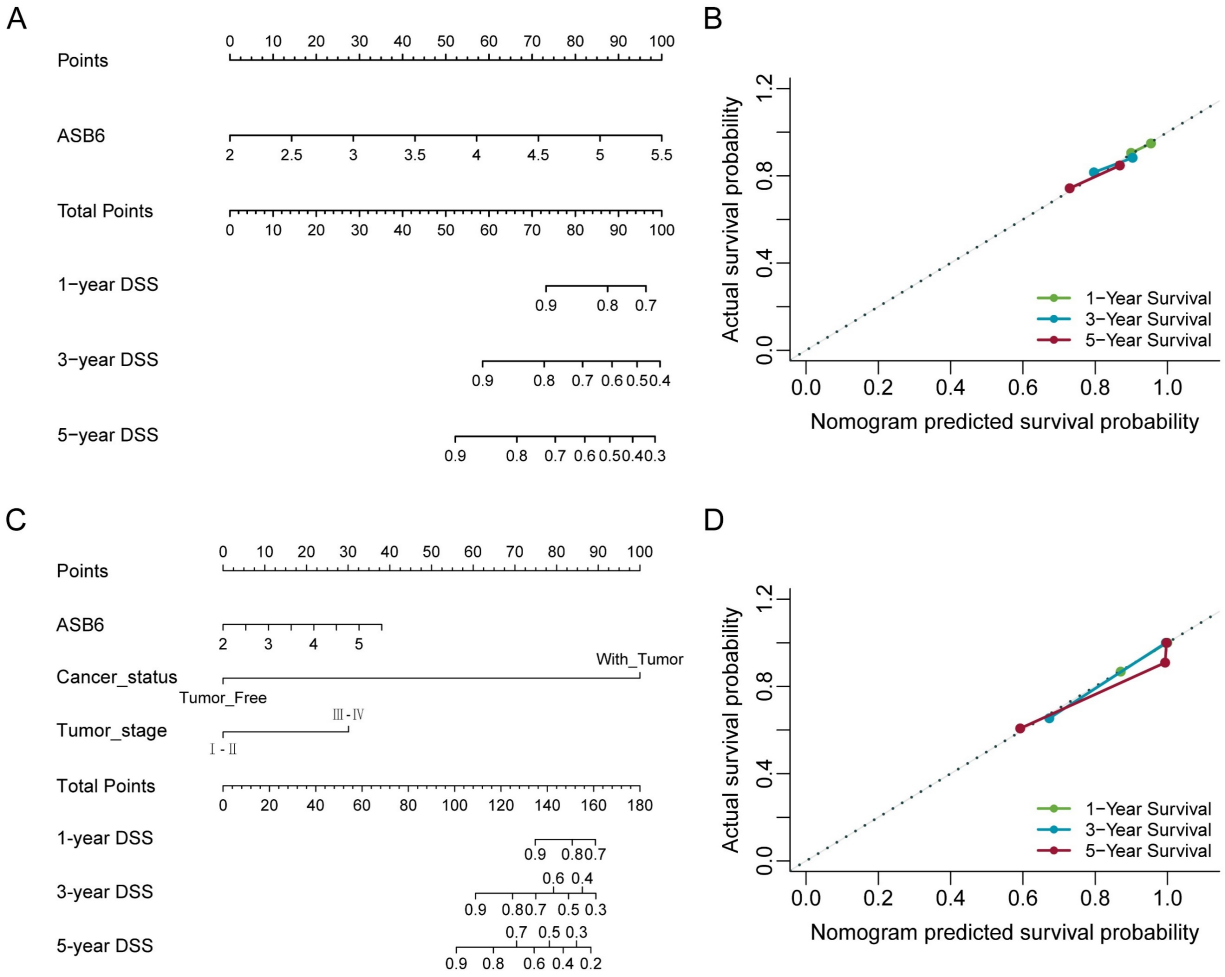
Nomogram for calculating risk score and predicting disease-specific survival (DSS) probability. (A, C) Postoperative prognostic nomogram for colorectal cancer patients was established based on ASB6 expression levels, cancer status, tumor stage; (B, D) Calibration curves of the nomogram were plotted to compare predicted and actual disease-specific survival (DSS) proportions at 1 year, 3 years, and 5 years. The x-axis represents the nomogram-predicted DSS probability, and the y-axis represents the actual DSS proportion.

The results of the univariate and multivariate Cox analyses showed that cancer status and ASB6 expression level were significantly associated with overall survival (OS). Meanwhile, cancer status, tumor stage, and ASB6 expression level were significantly correlated with disease-specific survival (DSS) (Tables 1, 2). To further elucidate the prognostic value of ASB6 in colorectal cancer, we constructed nomograms using the aforementioned independent prognostic factors associated with OS and DSS (Figure 4, 5). The TNM staging system serves as a classical standard for clinical diagnosis and prognosis. Therefore, we also constructed prognostic nomograms and calculated concordance index (C-index) values to assess their predictive accuracy according to the TNM staging (Supplementary Figure S1). The total score for each variable in the nomograms predicted 1-year, 3-year, and 5-year OS and DSS rates. Our analysis revealed that the nomograms constructed based on the variables from the multivariate analysis results (p < 0.05) (referred to as the New-nomogram) for both OS and DSS had better predictive performance than those based on the TNM staging (referred to as the TNM-nomogram). Specifically, the New-nomogram for OS demonstrated a c-index of 0.811 (95% CI 0.771-0.850), outperforming the TNM-nomogram, which had a c-index of 0.728 (95% CI 0.698-0.758). Similarly, the New-nomogram for DSS demonstrated a c-index of 0.934 (95% CI 0.922-0.947), surpassing the TNM-nomogram's c-index of 0.801 (95% CI 0.77-0.832). However, the nomogram constructed based solely on the expression of ASB6 (referred to as the ASB6-nomogram) had a slightly weaker predictive accuracy compared to the two aforementioned nomograms, with the c-index for the OS and DSS nomograms being 0.626 (95% CI 0.591-0.660) and 0.636 (95% CI 0.594-0.679), respectively. In addition, we compared the predictive abilities (c-index) of the three nomograms at 1-year, 3-year and 5-year timepoints, and the New-nomogram showed better performance at all three time points (Supplementary Table S1-2).

### ASB6 methylation and CNA profiles in COAD

**Figure 6:**
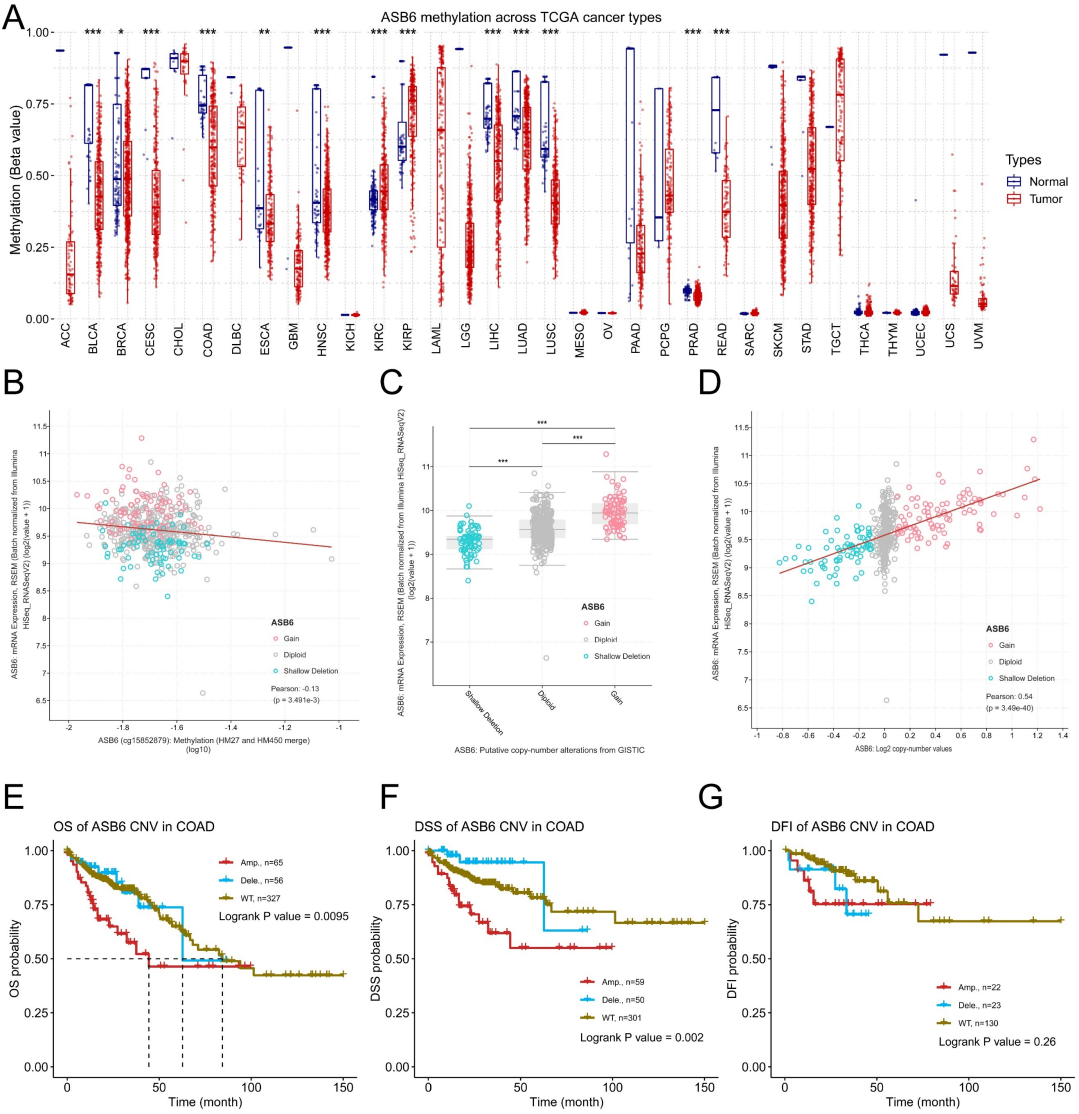
DNA copy number amplification and decreased methylation levels result in the upregulation of ASB6 in COAD. (A) The methylation status of ASB6 in pan-cancer; (B) The correlation between mRNA expression levels and DNA methylation of ASB6; (C) The mRNA expression levels of ASB6 in the copy number variation (CNV) subgroups; (D) The correlation between the mRNA expression levels and the copy number variation (CNV) values of ASB6. (E-G) Survival analysis of ASB6 in the copy number variation (CNV) subgroups; (*p < 0.05; **p < 0.01; ***p < 0.001; ****p < 0.0001; ns, Not Significant).

To further explore the potential mechanisms driving the upregulation of ASB6 in colorectal cancer, we analyzed its methylation levels through the GSCA database. Notably, the methylation levels of ASB6 were significantly decreased in various tumors, including BLCA, BRCA, CESC, COAD, ESCA, HNSC, LIHC, LUAD, LUSC, PRAD, and READ (Figure 6A). Furthermore, the methylation level of ASB6 was negatively correlated with its mRNA expression level (Figure 6B). Additionally, ASB6 copy number amplification was significantly associated with overexpression of ASB6 mRNA in colorectal cancer (Figure 6C, D). Subsequent survival analysis of ASB6 copy number alteration (CNA) subgroups (Amp, Dele and WT) in colorectal cancer patients revealed that patients with ASB6 copy number amplification (Amp) had worse OS, DSS and DFI compared to those with wild-type (WT) and deletion (Dele) ASB6 (Figure 6E-G).

### Correlation between ASB6 expression and immune infiltration or immunotherapy response in COAD

**Figure 7:**
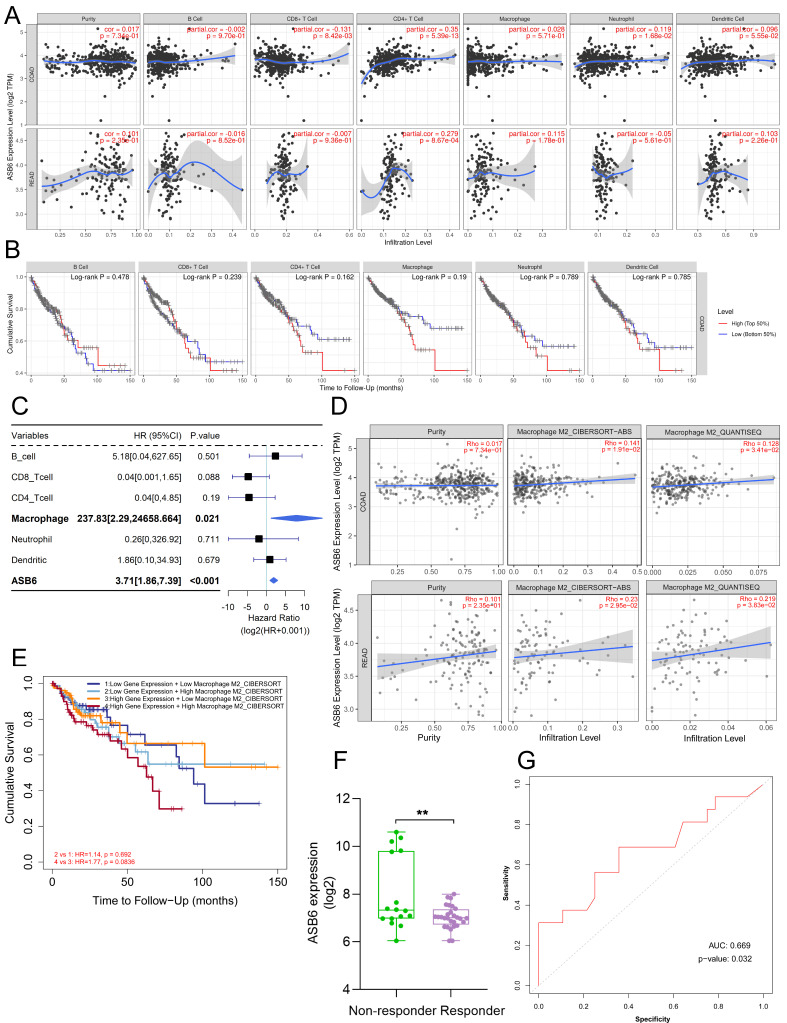
Correlation between ASB6 expression levels and immune infiltration levels in COAD and READ. (A) Analyzing the correlation between gene expression levels and immune infiltration levels using the TIMER database. (B) Survival analysis of Immune infiltration level. (C) Forest plot showing the correlation between immune cell scores and survival. (D) Using different algorithms (CIBERSORT or QUANTISEQ) to analyze the correlation between the expression of ASB6 and Macrophage M2 subtype. (E) Performing survival analysis on ASB6 gene expression and Macrophage M2 scores (high/low groups) using the TIMER database in COAD. (F) Using the ROCplotter online website to analyze the expression levels of ASB6 in responders and non-responders to anti-PD-L1 immunotherapy. (G) The ROC curve represents the predictive accuracy of anti-PD-L1 immunotherapy response based on ASB6 expression levels.

We utilized the TIMER database to analyze the correlation between ASB6 expression levels and immune cell infiltration. The results revealed that the expression of ASB6 was significantly negatively correlated with CD8+ T cells, and significantly positively correlated with CD4+ T cells and neutrophils in COAD (Figure 7A). ASB6 expression was significantly positively correlated with CD4+ T cells in READ (Figure 7A). Survival analysis of immune cell levels in colorectal cancer showed that higher levels of immune cells, such as CD4+ T cells or macrophages, were associated with worse overall survival (Figure 7B). Additionally, the results from univariate Cox analysis suggested that both macrophages and ASB6 were identified as risk factors in colorectal cancer (both p-values < 0.05) (Figure 7C). M2 Macrophage has been implicated in promoting tumor development 39, 40. Thus, we further analyzed the correlation between ASB6 and M2 Macrophage using both the CIBERSORT-ABS and QUANTISEQ databases. The analysis results from both databases showed a significant positive correlation between ASB6 expression levels and M2 Macrophage levels in both COAD and READ (both p-values < 0.05) (Figure 7D). Moreover, the combined survival analysis of ASB6 expression and M2 Macrophage levels also revealed that patients with high ASB6 expression and high M2 Macrophage levels exhibited shorter overall survival in colorectal cancer (p = 0.083) (Figure 7E). Furthermore, we evaluated the predictive value of ASB6 for tumor immune therapy response using the ROC Plotter database (https://www.rocplot.org/) 41. The results revealed that colorectal cancer patients with high ASB6 expression were less sensitive to anti-PD-L1 immunotherapy, with a predictive AUC value of 0.669 (p = 0.032) (Figure 7F, G).

### Enrichment analysis

**Figure 8:**
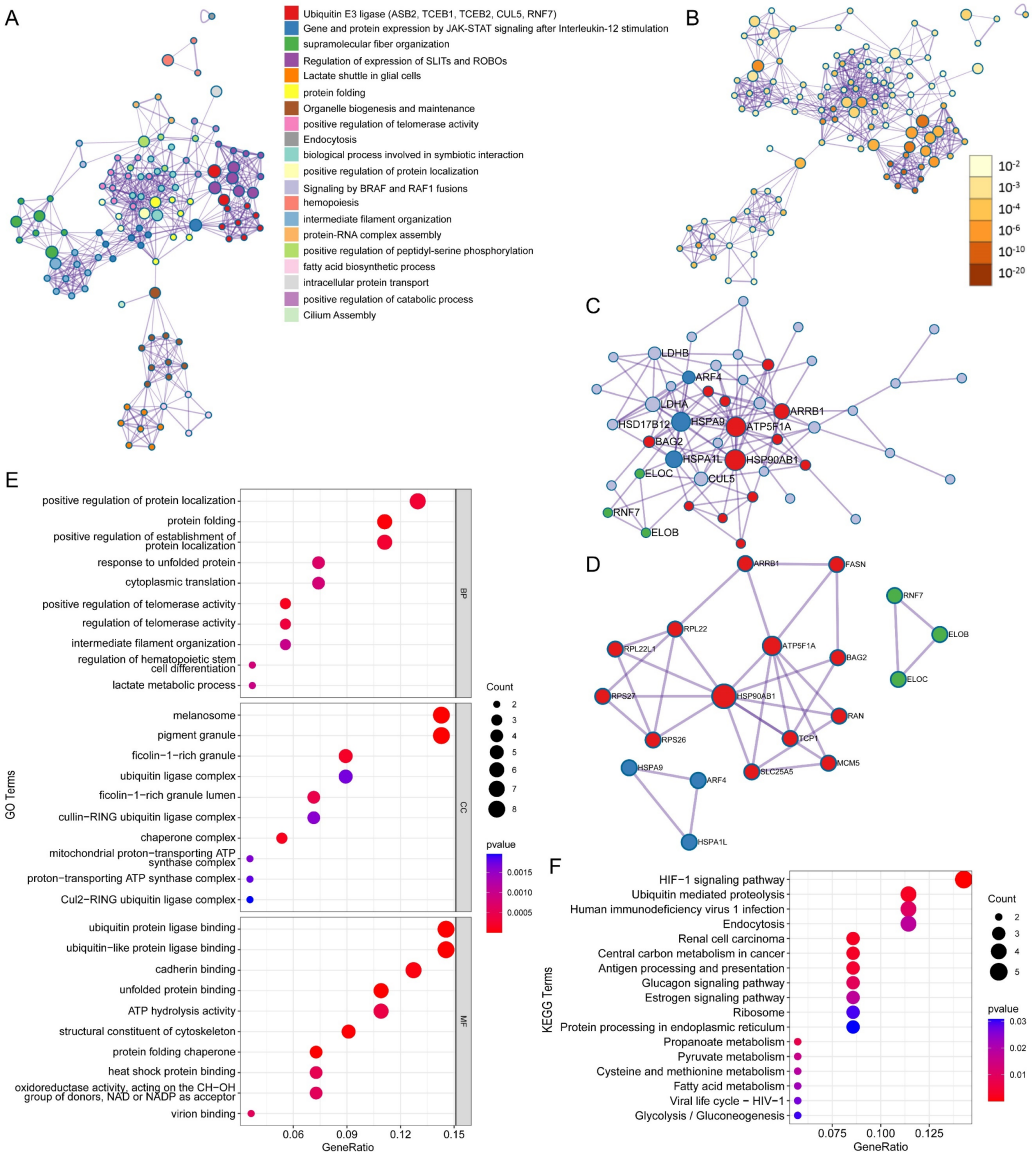
Protein-protein interaction (PPI) network and pathway enrichment analysis for ASB6 interacting proteins. (A) Different pathways are shown in various colors, colored by cluster ID. (B) P-values for the corresponding pathways in (A). (C) Protein-protein interaction network diagram. (D) MCODEs identification in the PPI network. (E, F) GO and KEGG functional enrichment analysis.

ASB6, an E3 ubiquitin ligase, can mediate substrate degradation through the ubiquitin-proteasome system, thereby regulating substrate stability and function. Considering the significant upregulation of ASB6 in colorectal cancer tissues, we identified 56 proteins that interact with ASB6 in colorectal cancer cells through immunoprecipitation-mass spectrometry analysis (Supplementary Table S1). To explore the potential functions of ASB6 in colorectal cancer, we used Metascape to construct a protein-protein interaction (PPI) network for these ASB6-interacting proteins and performed GO and KEGG enrichment analysis. The results showed that positive regulation of protein localization, protein folding, positive regulation of establishment of protein localization, response to unfolded protein, and cytoplasmic translation localization (Top 5) were significantly enriched in GO BP terms (Figure 8E). The top 5 significantly enriched KEGG pathways were HIF-1 signaling pathway, Ubiquitin mediated proteolysis, Human immunodeficiency virus 1 infection, Endocytosis, and Renal cell carcinoma (Figure 8F). The top ten results of GO enrichment analysis, including biological process (BP), cellular component (CC), and molecular function (MF) are shown in Figure 8E. Top 15 KEGG enrichment analysis results are shown in Figure 8F. Notably, the enriched GO and KEGG pathways were related to response to unfolded protein and protein processing in the endoplasmic reticulum, respectively, so we speculated that ASB6 may regulate endoplasmic reticulum function. The PPI network and MCODE components are shown in Figures 8A-D.

### ASB6 promotes cell migration

**Figure 9:**
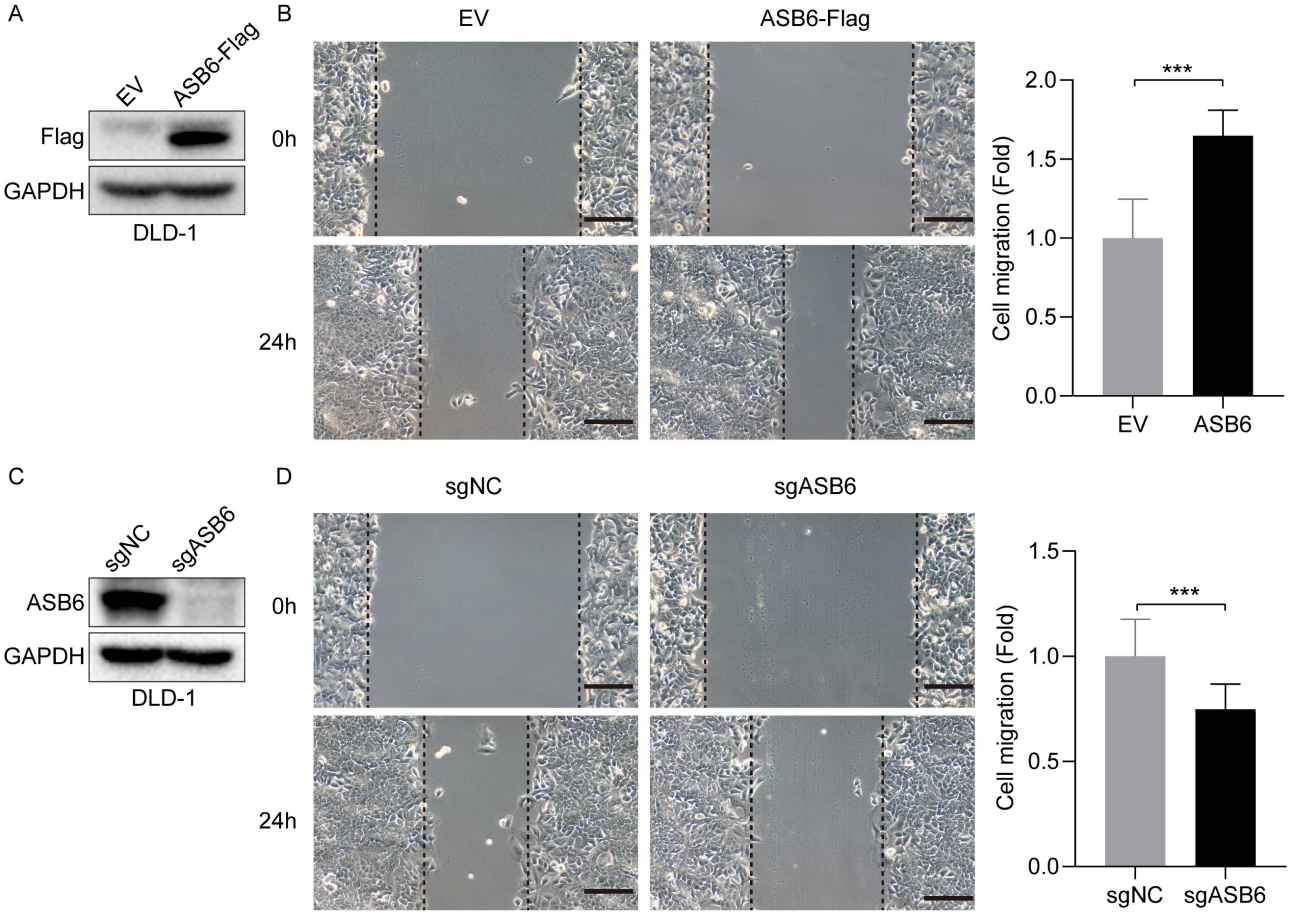
ASB6 promotes the migration of colorectal cancer cells. (A, C) The effect of overexpression or knockout of ASB6 was determined by Western blots. (B, D) Wound healing assays indicated that overexpression of ASB6 enhanced the migration ability of colorectal cancer cells, knockout of ASB6 suppressed the migration ability of colorectal cancer cells. Scale bar = 100 μm. (*p < 0.05; **p < 0.01; ***p < 0.001; ****p < 0.0001; ns, Not Significant).

As shown in Figure 3, the expression level of ASB6 was significantly upregulated in colorectal cancer tissues with lymphatic invasion and distal metastasis. To investigate whether ASB6 facilitates colorectal cancer progression, we first established stable DLD-1 colorectal cancer cell lines with overexpression or knockout of ASB6, then performed Western blot analysis to confirm the stable overexpression and knockout of ASB6 in these cell lines (Figure 9A, C). We then conducted wound healing assays to investigate the effect of ASB6 on colorectal cancer cell migration. The results showed that overexpression of ASB6 significantly enhanced DLD-1 cell migration (Figure 9B), whereas knockout of ASB6 significantly inhibited DLD-1 cell migration (Figure 9D).

## Discussion

The ubiquitin-proteasomal system (UPS), typically consisting of ubiquitin, ubiquitin-related enzymes, and the 26S proteasome, is one of the ways that regulates protein homeostasis. The UPS ubiquitinates target proteins through coordinated enzymatic reactions, which promotes their degradation via the proteasome pathway 42. The ubiquitination process relies on a three-step cascade of enzymatic reactions (E1, E2, and E3), among which E3 ubiquitin ligases play a crucial role by directly recognizing substrates during ubiquitination 16, 43.

Based on the structural similarities and ubiquitination domains of E3 ubiquitin ligases, the E3 ubiquitin ligase family can be divided into three main classes: the really interesting new gene (RING) finger domain-containing E3s, the homologous to E6-AP C-terminal (HECT) E3s, and the RING-in-between-RING (RBR) E3s 44, 45. Among them, the RING-type E3s are the most abundant class. The Cullin-RING E3 ubiquitin ligase (CRL) subgroup is responsible for up to 20% of ubiquitin-dependent protein degradation in cells 46. Structurally, CRLs complexes consist of four basic subunits: a Cullin scaffold, RING-finger proteins, adaptor proteins, and substrate recognition proteins 47. Within the CRL subgroup, SCF-type E3s are the most well-known class, utilizing Cullin1 as the scaffold protein, RBX1 as the RING-finger protein, SKP1 as the adaptor protein, and F-box proteins as the substrate receptor proteins 48. Similar to SCF-type E3s, the ECS (Elongin-Cullin-SOCS-box protein) E3 ubiquitin-protein ligase complex is also formed by four proteins, including ElonginB/C, Cullin5 (or Cullin2), RBX2 (or RBX1), and the substrate recognition receptor proteins 28, 43. Since E3 ubiquitin ligases determine substrate specificity, their dysregulation is associated with many human diseases, including cancers. Therefore, E3 ubiquitin ligases represent an attractive and important class of drug targets in cancer therapy. In our study, we systematically analyzed the correlation between E3 ubiquitin ligase and deubiquitinating enzyme genes and the prognosis of colorectal cancer patients, and identified ASB6 as a key prognostic gene. ASB6 is one of the 18 members of the ankyrin repeat-containing SOCS box protein family (ASBs), characterized by a conserved SOCS box motif and a variable number of ankyrin repeats 49. ASB6 interacts with ElonginB/C-Cullin5-RBX2 via the SOCS box motif to form an ECS E3 ubiquitin-protein ligase complex to exert its functions. We identified proteins interacting with ASB6 by co-immunoprecipitation-mass spectrometry analysis and also identified corresponding proteins of the ECS complex. In addition, we analyzed the mRNA expression levels of ASB6 in pan-cancer and found that ASB6 is significantly upregulated in many tumors. Survival analysis also showed that ASB6 is significantly associated with overall survival (OS), progression-free interval (PFI), disease-specific survival (DSS) and disease-free interval (DFI) in various cancers, including colorectal cancer. In addition, the protein level of ASB6 was significantly upregulated in colorectal cancer tissues, and colorectal cancer patients with high ASB6 expression had worse prognosis. Further analysis of TCGA clinical samples revealed that ASB6 mRNA expression levels were also significantly correlated with lymph node invasion, distal metastasis and other adverse events in colorectal cancer patients. In vitro cell experiments also indicated that ASB6 overexpression enhanced, while ASB6 knockout weakened, the migration ability of colorectal cancer cells. We hypothesize that ASB6 may act as an oncogene, promoting tumorigenesis and development. The results of the two aforementioned studies 31, 32 also further support our hypothesis. Univariate and multivariate analyses also showed that ASB6 can serve as an independent prognostic factor for OS and DSS in colorectal cancer. Furthermore, to explore the prognostic value of ASB6 in colorectal cancer, we constructed nomograms with independent risk factors that were significantly associated with OS and DSS in multivariate analysis. The results showed that the nomogram (New-nomogram) model had better predictive performance, especially for the DSS nomogram model (c-index value = 0.934). Compared with the traditional TNM staging nomogram, the New-nomogram showed better predictive ability at the 1-year, 3-year and 5-year timepoints for both OS and DSS. Based on this, our New-nomogram model has the potential to serve as a supplementary or alternative prognostic model to the TNM staging nomogram for colorectal cancer patients. These data suggest that ASB6 may be a promising prognostic marker and therapeutic target for colorectal cancer. Our study also provides a new model for further improving the prognosis of CRC patients.

In cancers including colorectal cancer, E3 ubiquitin ligases exhibit abnormalities in promoter methylation and copy number variation (CNV), which affect the regulation of E3 ubiquitin ligases, disturb cancer-related pathways, and promote tumorigenesis 50. To explore the reasons for the abnormal expression of ASB6 in tumors, we conducted an analysis from the perspective of epigenetics. Methylation data analysis from TCGA revealed a significant downregulation of methylation levels of ASB6 in various cancers, including colorectal cancer. Furthermore, the methylation levels of ASB6 were significantly negatively correlated with its mRNA expression levels. CNV analysis also showed that ASB6 copy number amplification was significantly associated with overexpression of ASB6 mRNA in colorectal cancer. More importantly, colorectal cancer patients with abnormal ASB6 CNV (Amp) had worse OS, DSS and DFI. These results indicate that abnormal methylation and CNV of ASB6 may play crucial role in the progression of colorectal cancer.

E3 ubiquitin ligases play a crucial role in regulating the tumor microenvironment and influencing tumor immunotherapy 51. For example, MDM2, a clinically highly investigated E3 ubiquitin ligase target for cancer treatment, promotes tumor growth and progression by mediating the ubiquitin-dependent degradation of the tumor suppressor p53 17. Later, researchers also found that targeting MDM2 has the effect of enhancing tumor immunotherapy. The small molecule inhibitor of MDM2, AMG-232, rendered tumor cells more susceptible to T cell-mediated killing in vitro 52. Another MDM2 inhibitor APG-115 synergized with PD1 inhibitors in a mouse model of cancer immunotherapy by promoting M1 macrophage polarization and T cell activation 53. Macrophages are one of the major populations of innate immune cells that infiltrate tumors and play important roles in regulating the tumor microenvironment and anti-tumor immunity 54. Traditionally, macrophages are classified into pro-inflammatory M1 and anti-inflammatory M2 types 55. Tumor-associated macrophages are often considered M2-type macrophages that suppress immune functions, induce angiogenesis, and promote tumor growth and metastasis by interacting with cancer cells 39, 40. Co-culture of M2 macrophages with colorectal cancer cells indicates that macrophages mediate the migration and invasion of colorectal cancer cells by inducing epithelial-mesenchymal transition and activating the PI3K/AKT signaling pathway 56. Univariate analysis demonstrated that macrophage infiltration level and ASB6 expression level are independent risk factors. Further analysis across multiple immune infiltration databases revealed that ASB6 is significantly positively correlated with M2 macrophages. Most importantly, colorectal cancer patients with both high ASB6 expression and high M2 macrophage infiltration have worse overall survival. Therefore, we hypothesize that ASB6 facilitates M2 macrophage polarization, thereby promoting colorectal cancer progression. In addition, colorectal cancer patients with high ASB6 expression were insensitive to anti-PD-L1 immunotherapy. Further, we speculate that ASB6 may play an important role in immunotherapy resistance, but the molecular mechanisms still need further study. Pathway analysis shows that ASB6 may play a crucial role through the response to unfolded protein pathway and protein processing in the endoplasmic reticulum pathway. Previous studies have reported that ASB6 promotes the stemness properties of oral squamous cell carcinoma cells by attenuating endoplasmic reticulum (ER) stress 30. ER stress is closely associated with the two pathways mentioned above, which further demonstrates the significant role of ASB6 in these two pathways. However, the molecular mechanisms still need further research. As a substrate recognition protein in the ECS E3 ubiquitin ligase complex, ASB6 may also promote tumorigenesis by ubiquitinating and degrading tumor suppressor proteins. Within the ASB6 interactome identified by immunoprecipitation-mass spectrometry, ATP5F1A was a core protein. ATP5F1A, a subunit of mitochondrial ATP synthase, facilitates oxidative phosphorylation 57. Tumor cells rely more on glycolysis, known as the "Warburg effect"^58^, ^59^. Inhibition of ATP5F1A has been shown to increase colorectal cancer cell proliferation, while its low expression correlates with poor prognosis in colorectal cancer patients 60. Collectively, we hypothesize that ASB6-mediated ubiquitination and degradation of ATP5F1A suppresses oxidative phosphorylation and enhance glycolysis to facilitate tumor progression.

Although we comprehensively analyzed the prognostic value of ASB6 in colorectal cancer, and identified ASB6 interacting proteins by immunoprecipitation-mass spectrometry analysis to construct a PPI network and perform pathway enrichment analysis, further in-depth research is still needed to elucidate the molecular mechanisms of ASB6 action. In particular, future studies should examine potential regulatory relationships between ASB6 and core proteins in the PPI network.

## Conclusions

In summary, we systematically analyzed the prognostic value of ASB6 in colorectal cancer. This work demonstrates that ASB6 is significantly overexpressed in various tumor tissues, including colorectal cancer. CRC patients with high ASB6 expression had worse DFI, DSS, OS, and PFI. Additionally, ASB6 expression levels positively correlate with lymph node invasion, distal metastasis, and M2-type macrophage infiltration. Further in vitro experiments also confirmed that ASB6 promotes the migration ability of colorectal cancer cells. The nomogram we constructed based on independent prognostic factors demonstrated superior predictive ability, especially for DSS. We showed that hypomethylation and copy number amplification of ASB6 in colorectal cancer are the causes of its abnormal overexpression. Patients with ASB6 copy number amplification had worse OS, DSS, and DFI. Taken together, this study demonstrates that ASB6 is an independent prognostic marker and potential therapeutic target in colorectal cancer.

## Supplementary Material

Supplementary figures and tables.

Supplementary Data 1. IP-MS analysis of ASB6-interacting proteins.

## Figures and Tables

**Table 1:** Univariate and Multivariate Cox regression analysis for overall survival (OS) of colorectal cancer patients

Variables	Univariate analysis		Multivariate analysis	
	HR (95% CI)	*p*	HR (95% CI)	*p*
Age (≥ 65 vs. < 65)	1.56(0.99,2.46)	0.055	-	-
Colon polyps present (Yes vs. No)	1.4(0.77,2.55)	0.266	-	-
History of colon polyps (Yes vs. No)	0.76(0.44,1.31)	0.329	-	-
KRAS mutation status (Yes vs. No)	1.42(0.61,3.29)	0.417	-	-
Lymphatic invasion (Yes vs. No)	2.16(1.39,3.35)	0.001	1.2(0.59,2.44)	0.623
pathologic_M (M1 vs. M0)	4.86(3.04,7.76)	<0.001	1.82(0.93,3.57)	0.079
pathologic_N (N1+ vs. N0)	2.63(1.73,4.01)	<0.001	0.82(0.28,2.43)	0.724
Pathologic_T (T3-T4 vs. T1-T2)	3.08(1.34,7.07)	0.008	1.37(0.56,3.34)	0.492
Cancer status (With tumor vs. Tumor free)	8.17(4.78,13.97)	<0.001	3.75(1.93,7.26)	<0.001
Venous invasion (Yes vs. No)	2.51(1.6,3.93)	<0.001	1.39(0.7,2.74)	0.347
Gender (Female vs. Male)	0.91(0.6,1.37)	0.639	-	-
Tumor stage (III-IV vs. I-II)	2.95(1.9,4.6)	<0.001	2.1(0.64,6.85)	0.220
ASB6 expression level (High vs. Low)	2.7(1.58,4.63)	<0.001	1.99(1.16,3.43)	0.013

**Table 2:** Univariate and Multivariate Cox regression analysis for Disease-specific survival (DSS) of colorectal cancer patients

Variables	Univariate analysis		Multivariate analysis	
	HR (95% CI)	*p*	HR (95% CI)	*p*
Age (≥ 65 vs. < 65)	1.05(0.61,1.82)	0.850	-	-
Colon polyps present (Yes vs. No)	1.55(0.72,3.38)	0.265	-	-
History of colon polyps (Yes vs. No)	1.02(0.53,1.96)	0.951	-	-
KRAS mutation status (Yes vs. No)	1.61(0.43,6.03)	0.479	-	-
Lymphatic invasion (Yes vs. No)	4.08(2.25,7.42)	**<0.001**	1.44(0.51,4.07)	0.490
pathologic_M (M1 vs. M0)	10.5(5.86,18.81)	**<0.001**	1.59(0.68,3.75)	0.285
pathologic_N (N1+ vs. N0)	4.68(2.55,8.58)	**<0.001**	0.49(0.15,1.61)	0.241
Pathologic_T (T3-T4 vs. T1-T2)	13.15(1.82,95.13)	**0.011**	2.24(0.28,18.12)	0.449
Cancer status (With tumor vs. Tumor free)	164.6(22.58,1200.07)	**<0.001**	65.56(8.44,509.36)	**<0.001**
Venous invasion (Yes vs. No)	4.57(2.61,8)	**<0.001**	1.48(0.59,3.72)	0.403
Gender (Female vs. Male)	0.87(0.51,1.48)	0.604	-	-
Tumor stage (III-IV vs. I-II)	7.25(3.53,14.87)	**<0.001**	5.06(1.22,20.96)	0.025
ASB6 expression level (High vs. Low)	4.5(2.09,9.68)	**<0.001**	2.39(1.1,5.19)	**0.028**

**Table 3:** Correlation between ASB6 expression levels and clinicopathological characteristics in colorectal cancer patients from the TCGA dataset.

		ASB6 Expression		
	Total(N=441)	High(N=133)	Low(N=308)	P-value
**Age (year)**				
< 65	168 (38.1%)	49 (36.8%)	119 (38.6%)	0.749
≥ 65	273 (61.9%)	84 (63.2%)	189 (61.4%)	
**Gender**				
Female	207 (46.9%)	56 (42.1%)	151 (49.0%)	0.212
Male	234 (53.1%)	77 (57.9%)	157 (51.0%)	
**Kras mutation status**				
No	26 (5.9%)	8 (6.0%)	18 (5.8%)	0.796
Yes	22 (5.0%)	8 (6.0%)	14 (4.5%)	
Unknown	393 (89.1%)	117 (88.0%)	276 (89.6%)	
**Lymphatic invasion**				
No	249 (56.5%)	60 (45.1%)	189 (61.4%)	**0.00665**
Yes	151 (34.2%)	57 (42.9%)	94 (30.5%)	
Unknown	41 (9.3%)	16 (12.0%)	25 (8.1%)	
**T stage**				
T1-T2	87 (19.7%)	24 (18.0%)	63 (20.5%)	0.723
T3-T4	353 (80.0%)	109 (82.0%)	244 (79.2%)	
Unknown	1 (0.2%)	0 (0%)	1 (0.3%)	
**N stage**				
N0	263 (59.6%)	75 (56.4%)	188 (61.0%)	0.398
N1+	178 (40.4%)	58 (43.6%)	120 (39.0%)	
**M stage**				
M0	327 (74.1%)	94 (70.7%)	233 (75.6%)	0.0849
M1	59 (13.4%)	25 (18.8%)	34 (11.0%)	
Unknown	55 (12.5%)	14 (10.5%)	41 (13.3%)	
**History of colon polyps**				
No	242 (54.9%)	72 (54.1%)	170 (55.2%)	0.947
Yes	133 (30.2%)	40 (30.1%)	93 (30.2%)	
Unknown	66 (15.0%)	21 (15.8%)	45 (14.6%)	
**Living status**				
Alive	349 (79.1%)	93 (69.9%)	256 (83.1%)	**0.00221**
Dead	92 (20.9%)	40 (30.1%)	52 (16.9%)	
**Stage**				
Ⅰ-Ⅱ	248 (56.2%)	68 (51.1%)	180 (58.4%)	0.351
Ⅲ-Ⅳ	182 (41.3%)	61 (45.9%)	121 (39.3%)	
Unknown	11 (2.5%)	4 (3.0%)	7 (2.3%)	
**Cancer status**				
Tumor Free	283 (64.2%)	75 (56.4%)	208 (67.5%)	0.0806
With Tumor	72 (16.3%)	26 (19.5%)	46 (14.9%)	
Unknown	86 (19.5%)	32 (24.1%)	54 (17.5%)	

## Abbreviations

CRC: Colorectal cancer
TCGA: the cancer genome atlas
ASB6: Ankyrin repeat and SOCS box protein 6
DFI: Disease-free interval
DSS: Disease-specific survival
OS: Overall survival
PFI: Progression-free interval
ACC: Adrenocortical carcinoma
BLCA: Bladder Urothelial Carcinoma
BRCA: Breast invasive carcinoma
CESC: Cervical squamous cell carcinoma and endocervical adenocarcinoma
CHOL: Cholangiocarcinoma
COAD: Colon adenocarcinoma
DLBC: Lymphoid Neoplasm Diffuse Large B-cell Lymphoma
ESCA: Esophageal carcinoma
GBM: Glioblastoma multiforme
HNSC: Head and Neck squamous cell carcinoma
KICH: Kidney chromophobe
KIRC: Kidney renal clear cell carcinoma
KIRP: Kidney renal papillary cell carcinoma
LGG: Brain Lower Grade Glioma
OV: Ovarian serous cystadenocarcinoma
MESO: Mesothelioma
LIHC: Liver hepatocellular carcinoma
LUAD: Lung adenocarcinoma
LUSC: Lung squamous cell carcinoma
PAAD: Pancreatic adenocarcinoma
PRAD: Prostate adenocarcinoma
PCPG: Pheochromocytoma and Paraganglioma
READ: Rectum adenocarcinoma
SARC: Sarcoma
SKCM: Skin Cutaneous Melanoma
LAML: Acute Myeloid Leukemia
TGCT: Testicular Germ Cell Tumors
THCA: Thyroid carcinoma
THYM: Thymoma
STAD: Stomach adenocarcinoma
UCEC: Uterine Corpus Endometrial Carcinoma
UCS: Uterine Carcinosarcoma
UVM: Uveal Melanoma
CNA: copy number alteration
