# Supplementary material for: ASB6 as an Independent Prognostic Biomarker for Colorectal Cancer Progression Involves Lymphatic Invasion and Immune Infiltration
Source: J Cancer. 2024 Mar 17;15(9):2712–30. doi: 10.7150/jca.93066 (PMC10988317; doi:10.7150/jca.93066)
Supplement: Supplementary file 1 — Supplementary figures and tables. [file jcav15p2712s1.pdf]

SUPPLEMENTARY INFORMATION

ASB6 as an Independent Prognostic Biomarker for Colorectal Cancer Progression Involves Lymphatic Invasion and Immune Infiltration

Qingyong Hu[†*], Yahui Chen[†], Qianru Zhou, Shanshan Deng, Bo Mu, and Jiancai Tang[*]

Institute of Basic Medicine and Forensic Medicine, North Sichuan Medical College, Nanchong, 637000, China

[†]These authors contributed equally to this work.

[*]Co-correspondence: Jiancai Tang and Qingyong Hu
Email: tangjiancai@nsmc.edu.cn (Jiancai Tang); huqy@nsmc.edu.cn (Qingyong Hu)

Supplementary Figures S1

Supplementary Table S1-2

Supplementary Data 1

Supplementary Figure S1

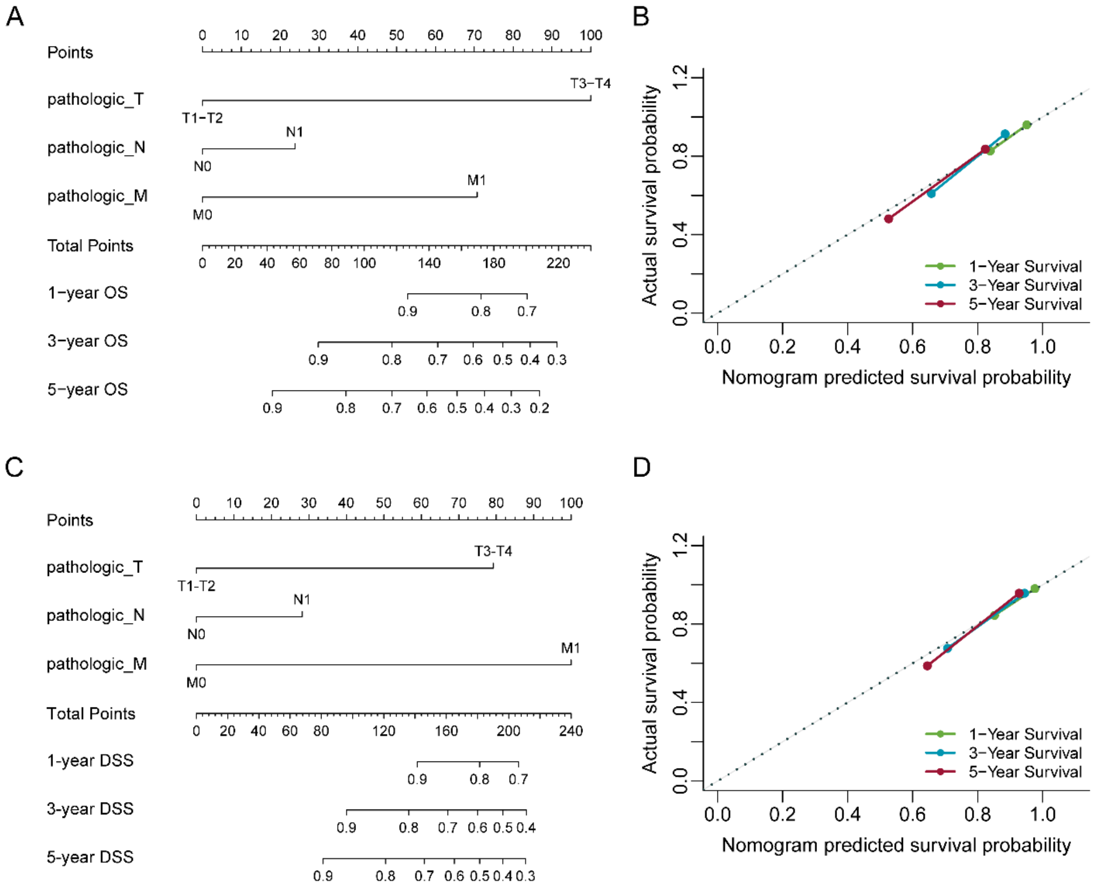

Supplementary Figure S1. Nomogram for calculating risk score and predicting the probabilities of overall survival (OS) or disease-specific survival (DSS). (A, C) Postoperative prognostic nomogram for colorectal cancer patients was established based on the TNM staging system; (B, D) Calibration curves for the nomogram were plotted to evaluate the congruence between the predicted and observed proportions of OS or DSS at 1, 3, and 5 years. The x-axis denotes the survival probability as predicted by the nomogram, while the y-axis indicates the actual observed survival proportion.

Supplementary Table S1. The predictive performance (c-index) of the nomogram constructed based on different variables for overall survival (OS).

Overall survival	TNM stage (TNM-nomogram)	ASB6 (ASB6-nomogram)	ASB6 + Cancer status +M stage (New-nomogram)
1-year	0.614(95%CI 0.561-0.668)	0.566(95%CI 0.509-0.623)	0.765(95%CI 0.701-0.830)
3-year	0.724(95%CI 0.692-0.757)	0.631(95%CI 0.594-0.668)	0.816(95%CI 0.773-0.858)
5-year	0.734(95%CI 0.703-0.764)	0.630(95%CI 0.594-0.665)	0.820(95%CI 0.780-0.860)

Supplementary Table S2. The predictive performance (c-index) of the nomogram constructed based on different variables for disease-specific survival (DSS).

Disease-specific survival	TNM stage (TNM-nomogram)	ASB6 (ASB6-nomogram)	ASB6 + Cancer status +Tumor stage (New-nomogram)
1-year	0.709(95%CI 0.654-0.764)	0.613(95%CI 0.549-0.678)	0.923(95%CI 0.892-0.954)
3-year	0.791(95%CI 0.759-0.824)	0.643(95%CI 0.598-0.687)	0.929(95%CI 0.915-0.944)
5-year	0.803(95%CI 0.772-0.834)	0.639(95%CI 0.595-0.682)	0.934(95%CI 0.921-0.947)

Supplementary Data 1. IP-MS analysis of ASB6-interacting proteins.
